# Supplementary material for: Chromatin state origins of uterine leiomyoma
Source: Nat Commun. 2025 May 8;16:4307. doi: 10.1038/s41467-025-59646-w (PMC12062214; doi:10.1038/s41467-025-59646-w)
Supplement: Supplementary file 1 — Supplementary Information [file 41467_2025_59646_MOESM1_ESM.pdf]

Supplementary figures

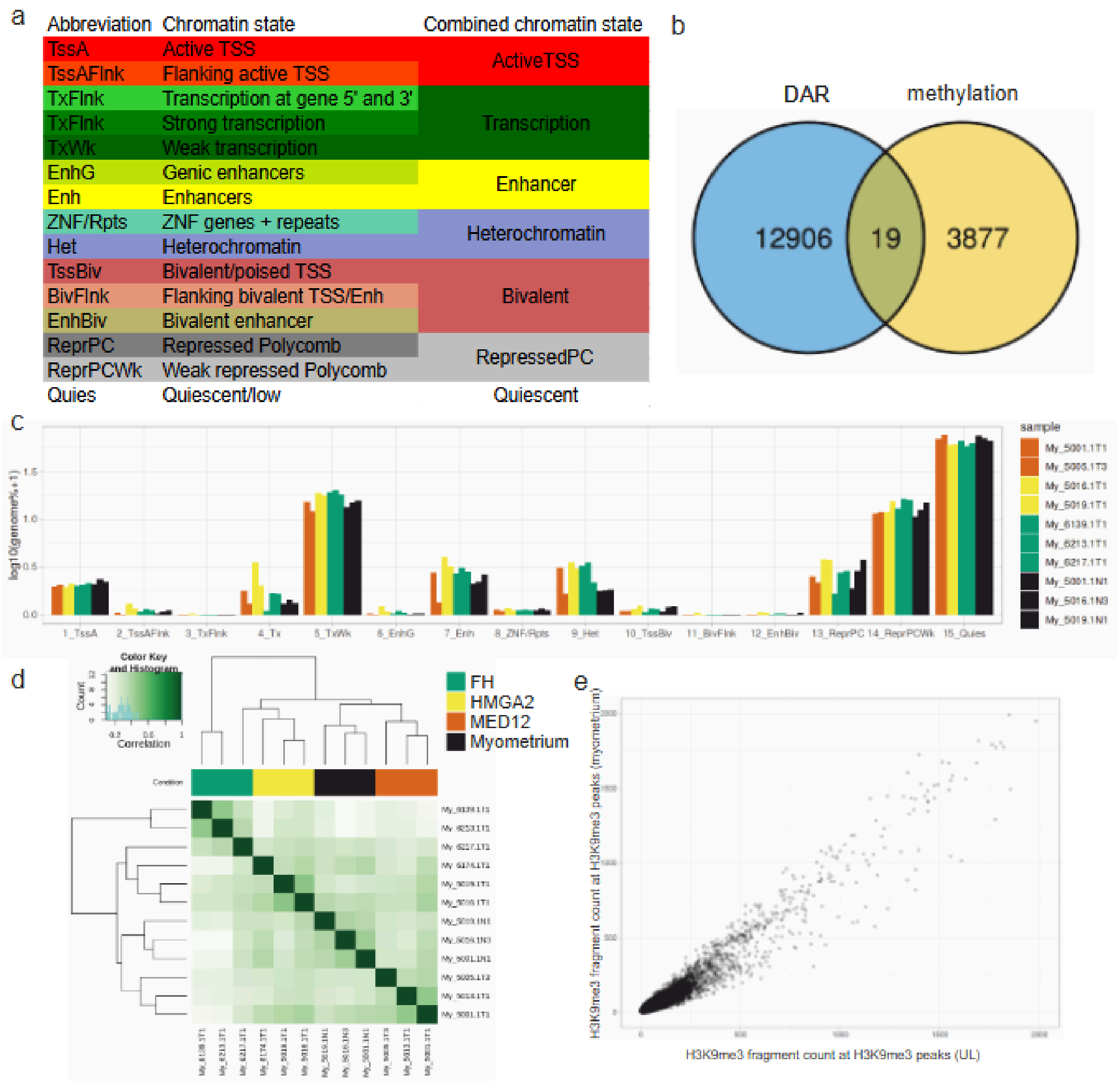

Supplementary Figure 1. Chromatin annotation of myometrium and UL. a. 15-state chromatin annotation and combination into 7 groups. b. Overlap of enhancers with DAR or hypermethylation in FH ULs. c. Sample-level annotations show similar amounts of each annotation. n=1 samples/subclass. d. DiffBind clustering of H3K9me3 ChIP-seq samples. e. H3K9me3 fragment counts at H3K9me3 peaks correspond between myometrium and UL. Data from one UL and one myometrium sample from the same patient.

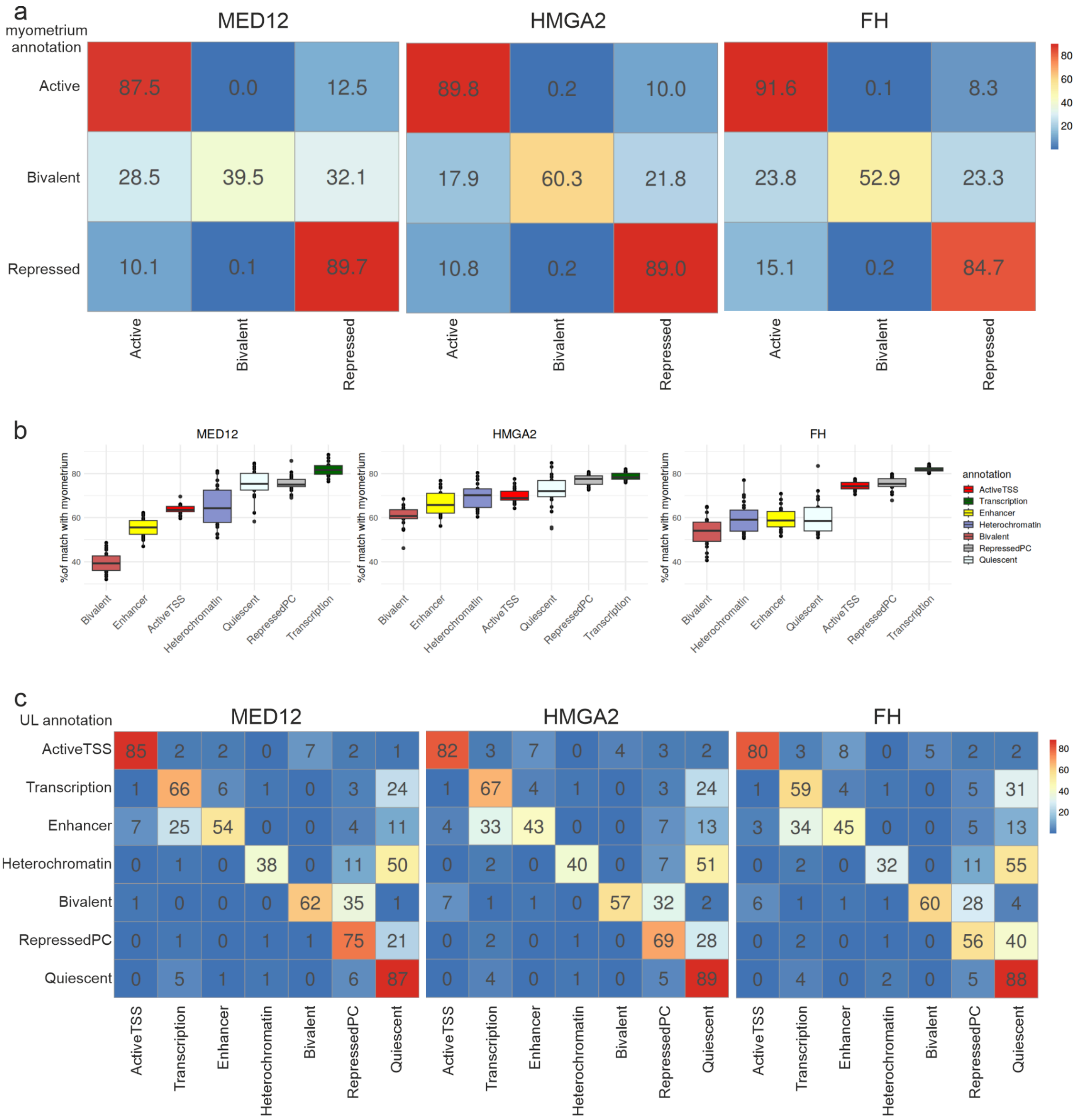

Supplementary Figure 2. Perturbation of bivalent regions in ULs. a. Correspondence of three annotation categories shows similarities of active and repressed states between myometrium and UL subclasses, but differences in bivalent regions. b. Bivalent regions have the lowest correspondence to myometrium annotation in ULs. Analysis done separately for each autosomal chromosome (n=22). Centre line, median; box limits, 25% and 75% quartiles; whiskers, 1.5 × interquartile range (IQR) past the quartiles. c. Correspondence of UL annotations in myometrium. Heterochromatin has the lowest correspondence to myometrium annotation in each UL subclass.

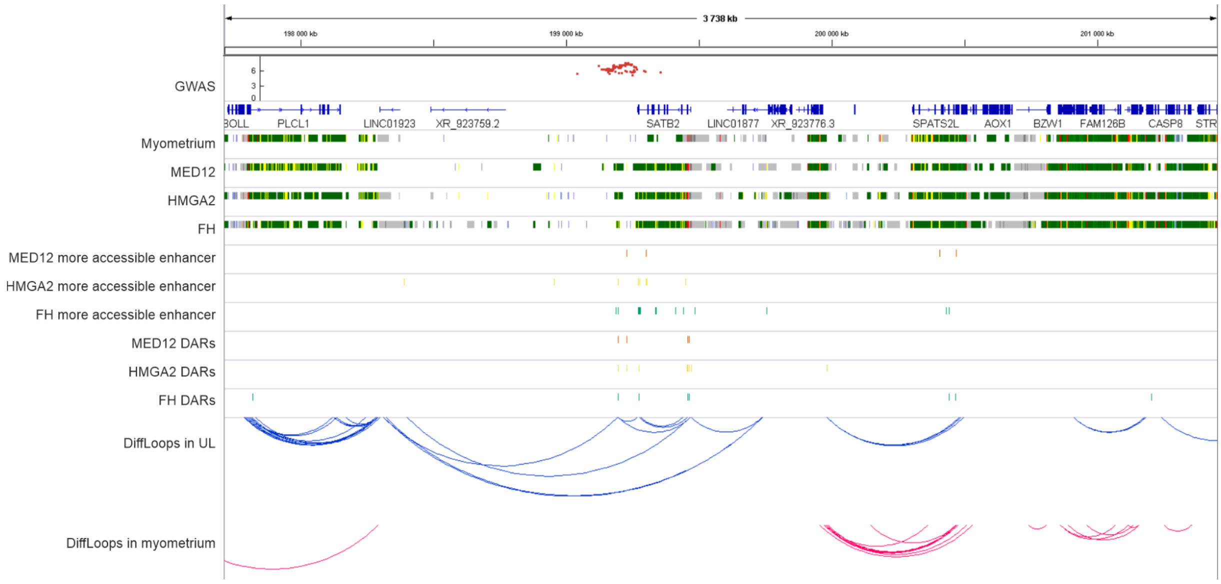

Supplementary Figure 3. *SATB2*-locus harbors many chromatin perturbations in ULs. Active chromatin states emerge in ULs compared to quiescent chromatin in myometrium. In accordance, more accessible UL enhancers and more accessible regions mapped to the region. Differential interactions connect GWAS signal to SATB2 promoter in ULs. See Supplementary Figure 1a for color definitions. Chromatin segmentations per subclass derived from 2-3 biological replicates/ChIP experiment/subclass. More accessible regions calculated with 4 ULs/subclass against 15 myometrium samples. Differential interactions derived from pooled data from 5 ULs and 5 myometrium samples.

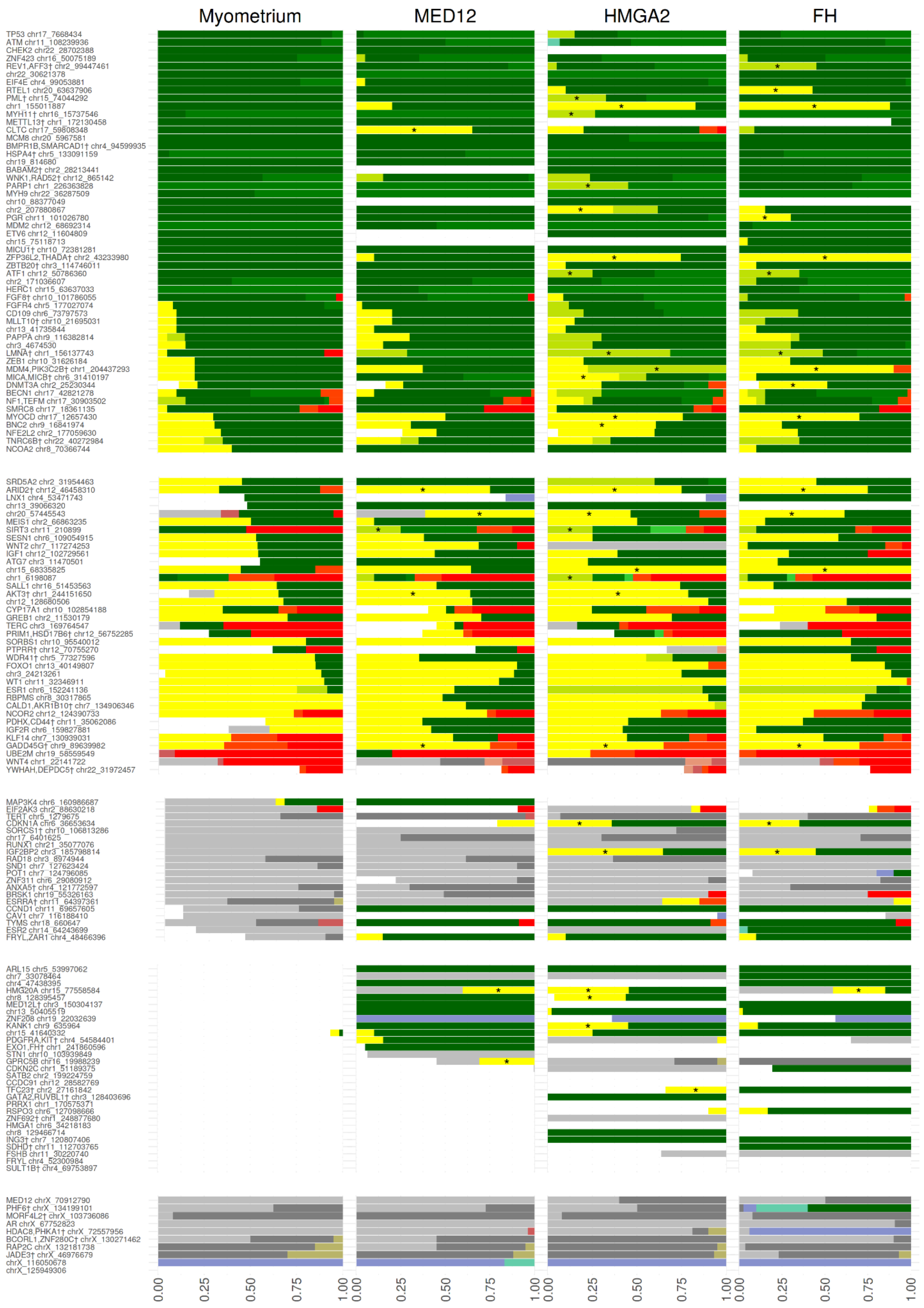

Supplementary Figure 4. GWAS regions (most significant SNP +2000bp flanks) annotated with myometrium and MED12, HMGA2 and FH UL chromatin annotations. GWAS regions can be divided into four categories based on their chromatin annotation in

myometrium: Transcription (n=53), Enhancer (n=37), Repressed polycomb (n=20) and Quiescent (n=29). In addition 10 regions map to chromosome X. See Supplementary Figure 1a for color definitions.

* Regions with at least 25% more enhancer annotation in UL compared to myometrium.

† Multiple genes and/or target gene is not clear at all.

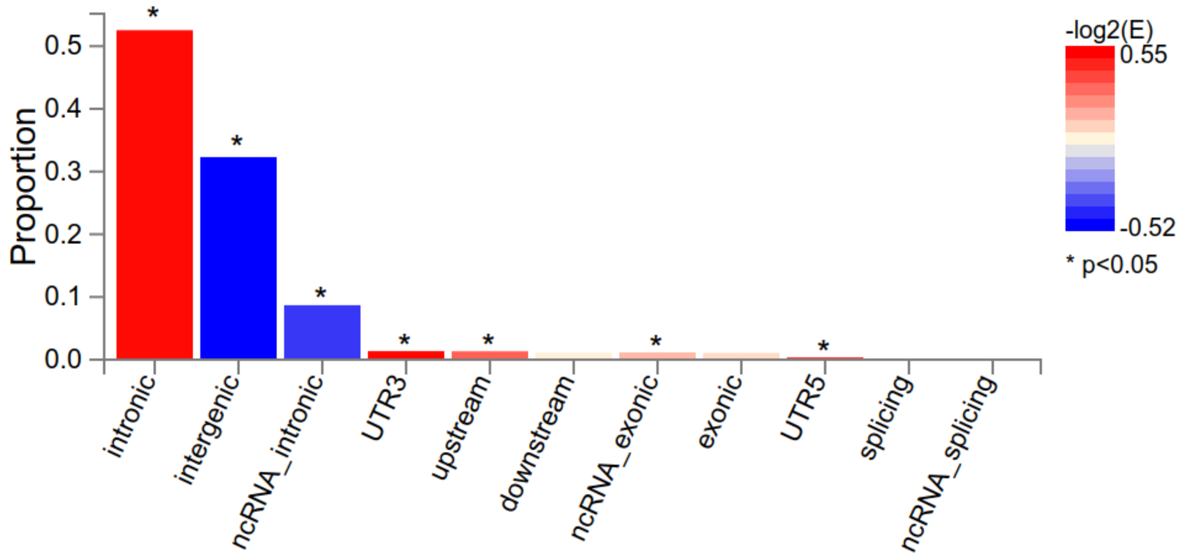

Supplementary Figure 5. FUMA functional consequence analysis of GWAS SNPs. Majority of the GWAS SNPs were positively enriched to regions that were annotated intronic, UTR3/5, upstream or non-coding RNA. Negative enrichment was predominantly in intergenic regions (N=31796 SNPs; two-sided Fisher's exact test). Exact P-values are given in the Source Data file.

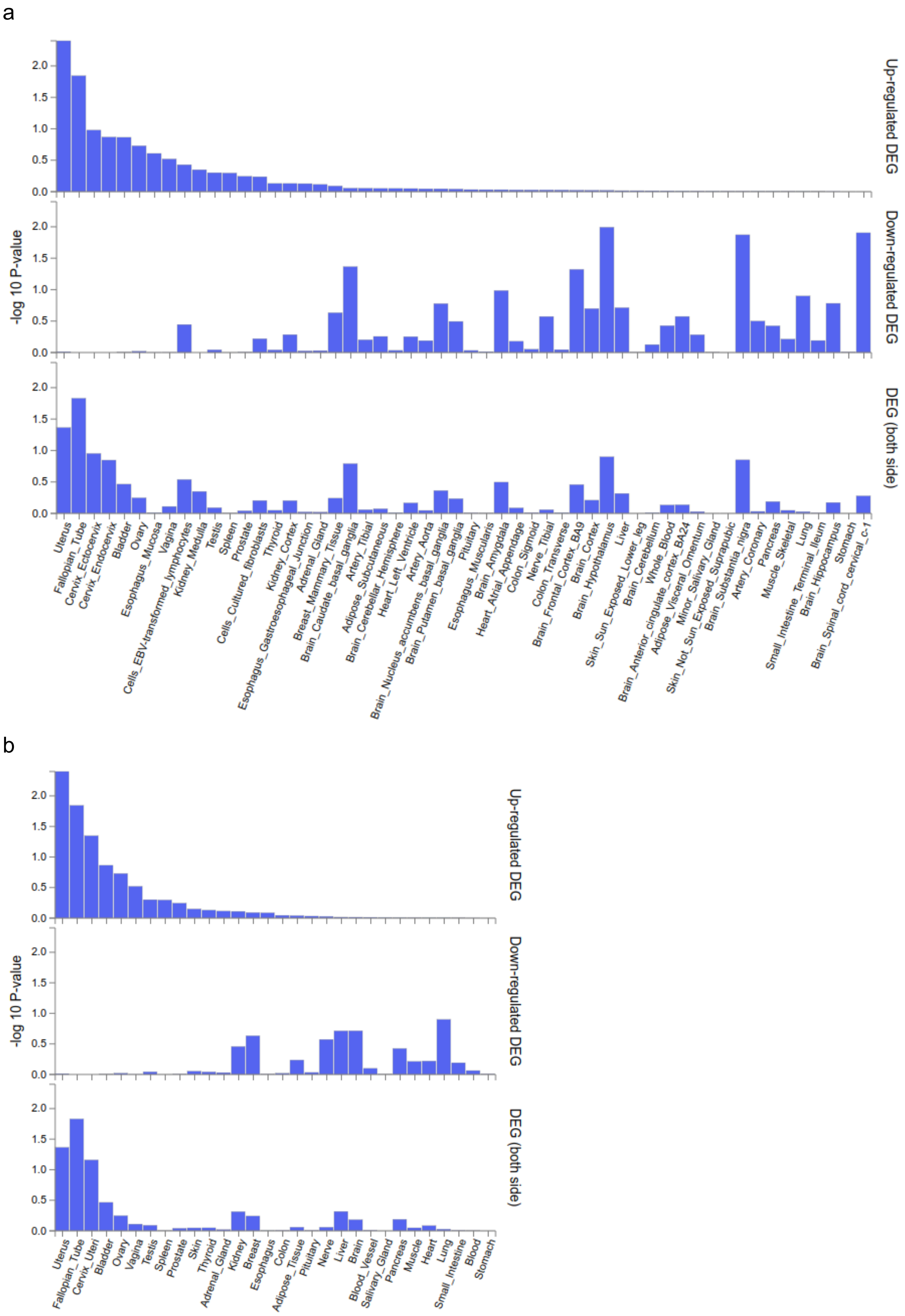

Supplementary Figure 6. FUMA differentially expressed genes (DEG) analysis of the 849 genes mapped based on GWAS SNPs. Two panels, each with three graphs showing

the enrichment for up-regulated DEGs, down-regulated DEGs and DEGs from both directions. Uterine tissue had the strongest enrichment for up-regulated genes among the 54 tissue types (a; top panel; N=849 genes) and among the 30 general tissue types (b; bottom panel; N=849 genes). P-values are from a one-sided hypergeometric test (FUMA). Exact P-values are given in the Source Data file.

a

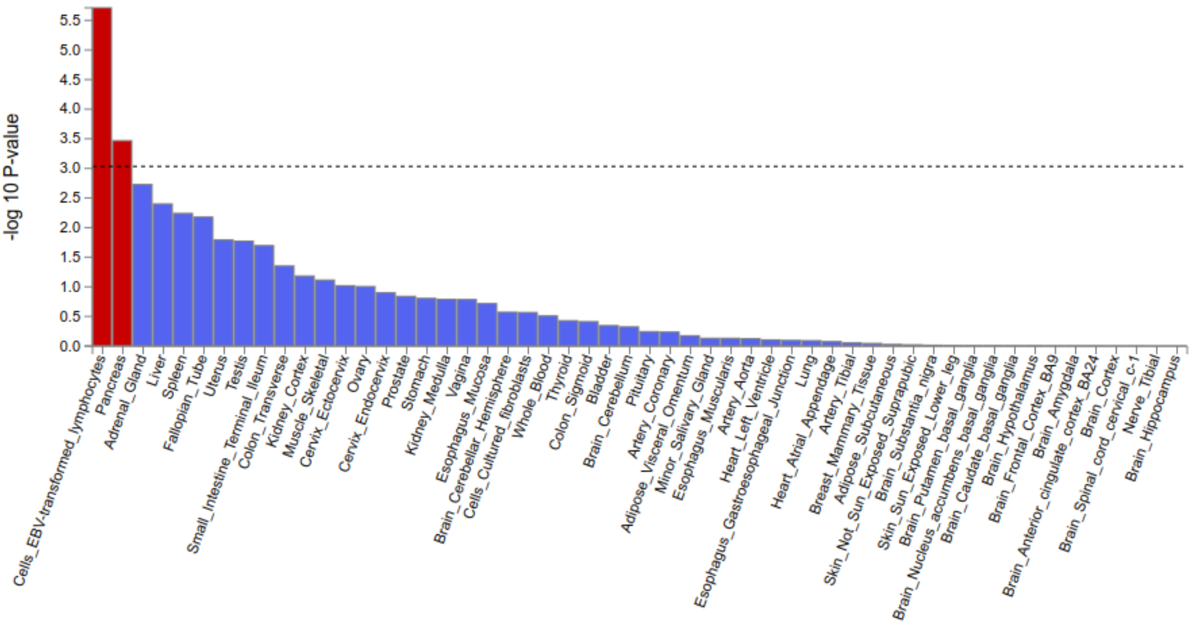

b

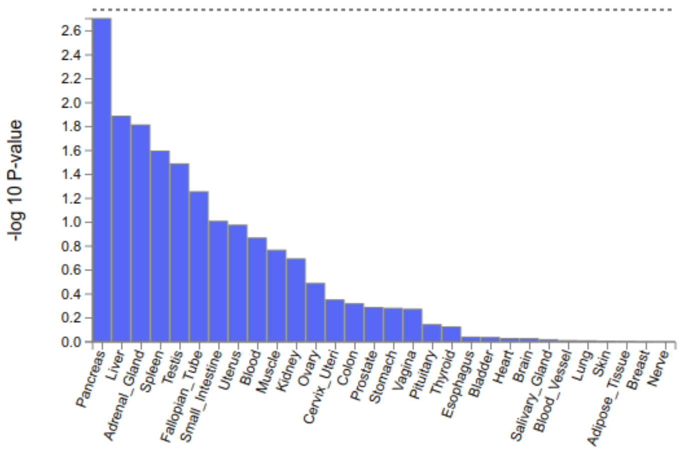

Supplementary Figure 7. MAGMA tissue expression analysis of the GWAS SNPs. Two panels which show the default MAGMA tissue expression analysis. Uterine tissue was found to rank seventh of the 53 tissue types analysed (a; top panel; N=16755 genes) and eight of the 30 general tissue types (b; bottom panel, N=16755 genes). P-values are from a one-sided MAGMA gene-property analysis test. Exact P-values are given in the Source Data file.

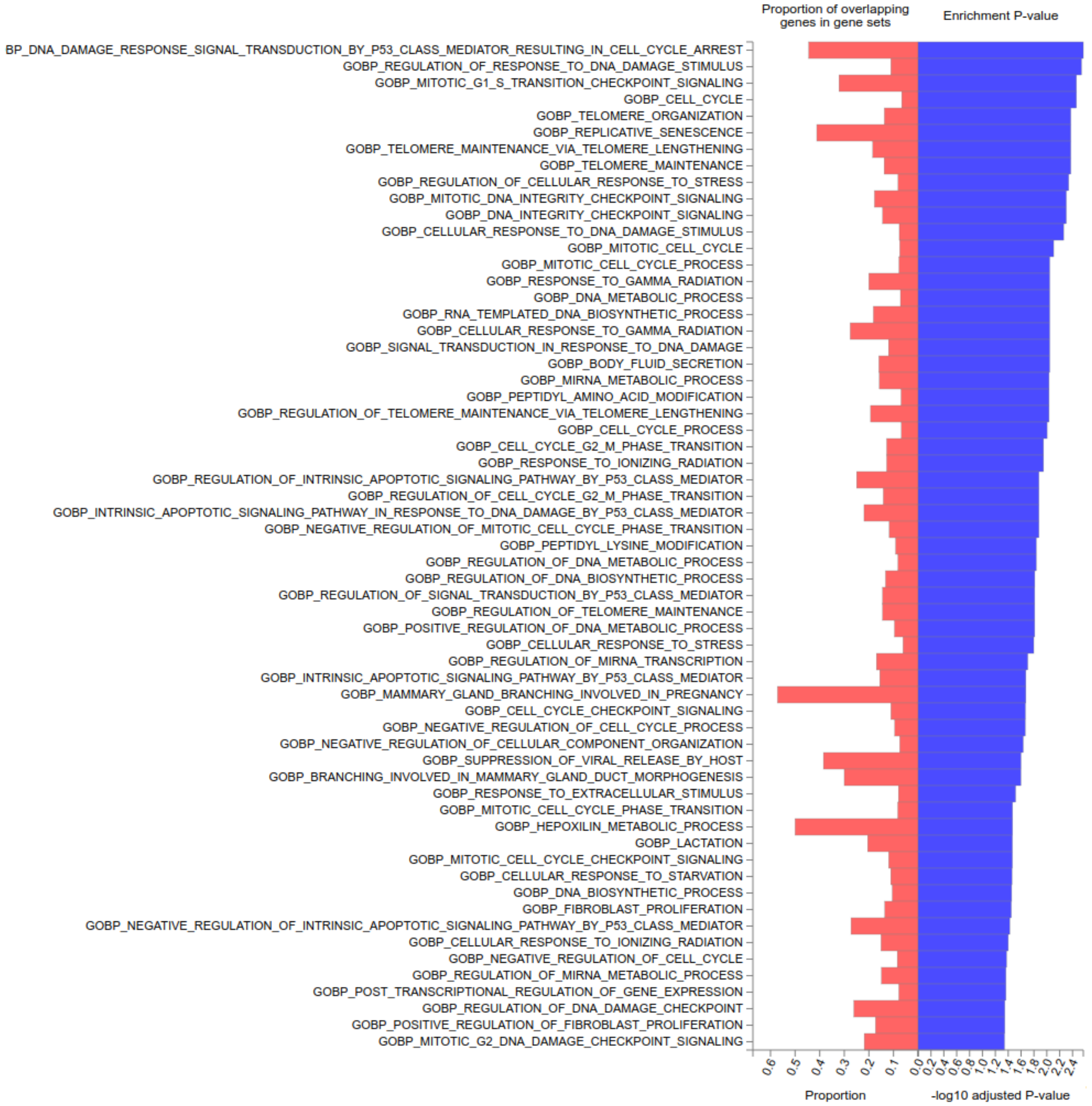

Supplementary Figure 8. FUMA gene-set enrichment. Gene-set enrichment analysis of 849 genes mapped based on GWAS SNPs. The figure shows all significant (FDR adjusted P<0.05, Benjamini-Hochberg adjusted one-sided hypergeometric test, N=849 genes) pathways enriched among GO biological processes. Blue: log-scaled FDR adjusted P-value; red: proportion of overlapping genes in each gene-set. Exact P-values are given in Supplementary Data 7.

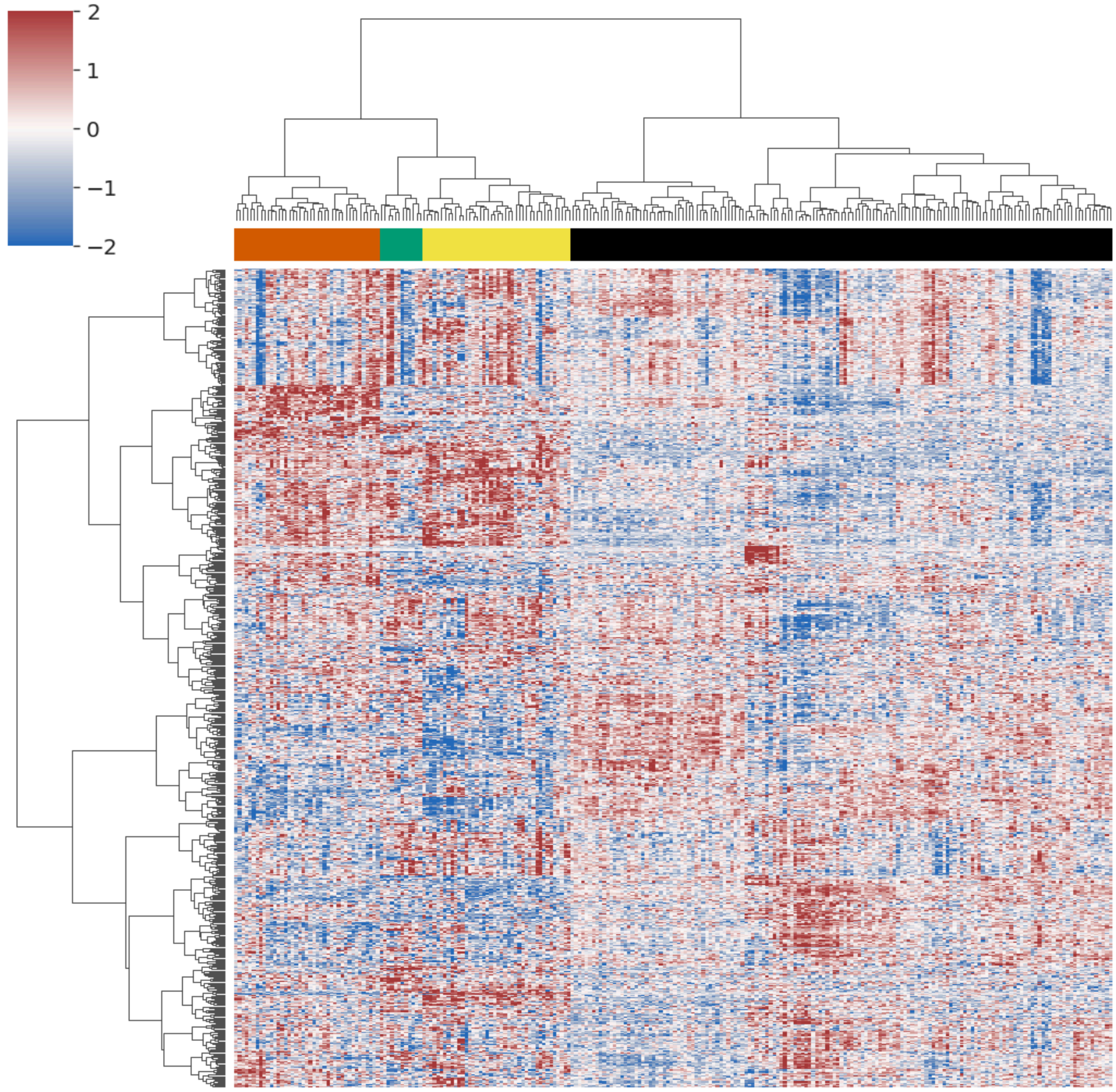

Supplementary Figure 9. FUMA gene-set separates myometrium and the three subclasses. Hierarchical clustering of the 849 genes identified by FUMA analysis of GWAS SNPs. Gene expression data from Berta *et al. Nature*'21 included 683/849 genes that passed their minimum expression threshold. Heatmap displays up-regulated (red) and down-regulated (blue) genes per sample. The column colors are as follows: black: myometrium (n=153), orange: MED12 (n=41), green: FH (n=12), yellow: HMGA2 (n=42).

Supplementary tables

Supplementary table 1. Antibodies used in ChIP-seq experiments

Target	Antibody	Used lot(s)	Dynabeads	Amount of antibody used
H3K4me1	Abcam ab8895	1017389-1	G	7ug
H3K4me3	Abcam ab8580	GR3275503-1, GR3264593-1	A	7ug
H3K27me3	Cell signaling 9733S	16, 19	G	20ul
H3K9me3	Active Motif 39062 /39161	1022004	G	10ul
H3K36me3	Diagenode C15410058	A.8889-001P	G	7ug
