# Supplementary material for: Chromatin state origins of uterine leiomyoma
Source: Nat Commun. 2025 May 8;16:4307. doi: 10.1038/s41467-025-59646-w (PMC12062214; doi:10.1038/s41467-025-59646-w)
Supplement: Supplementary file 2 — Description of Additional Supplementary Information [file 41467_2025_59646_MOESM2_ESM.pdf]

## Description of Additional Supplementary Files

**Supplementary Data 1 FinnGen banner**

Detailed author information for the author named FinnGen.

**Supplementary Data 2 ChromVAR motif accessibility deviations at myometrium accessible enhancers hypermethylated in FH ULs**

Mean motif accessibility deviation scores for FH ULs and myometrium samples at myometrium accessible enhancers hypermethylated in FH ULs. diffanalysis p value adjusted = adjusted p-value for the difference in deviation scores between FH ULs and myometrium samples. Two-sided t-test was used to derive p-values for differential deviations. Reported p-values are adjusted for multiple testing.

**Supplementary Data 3 Myometrium bivalent genes**

List of genes annotated as bivalent in myometrium. In addition, list of myometrium bivalent genes activated or repressed in UL subclasses. Gene expression values for UL subclass vs. myometrium samples are from Berta et al 2021. FDR: false discovery rate, log2FC: log2 Fold Change. Two-sided Wald test implemented in DESeq2 was used for statistical testing, p-values are corrected for multiple testing.

**Supplementary Data 4. More accessible enhancer target genes identified in at least two UL subclasses in all three analyses to map target genes.** Genes identified as a target for UL more accessible enhancers with all three methods used in at least two UL subclasses. Differential expression values for UL subclass vs. myometrium samples were obtained from Berta et al. 2021. FDR: false discovery rate, log2FC: log2 Fold Change.

**Supplementary Data 5 GWAS meta-analysis genome-wide significant associations to UL**

GWAS of altogether 66,075 UL cases and 537,502 female controls. A short-list of the most significant associations in an 1Mbp window that passed inverse-variance weighted fixed effects meta-analysis P-value < 5×10-8, including the summary statistics for each of the three cohorts, FINNGEN, UK Biobank (UKB) and Biobank Japan (BBJ). Genomic coordinates are in hg38. Allele-frequencies (AF) and effect sizes (BETA; two-sided mixed model logistic

regression) are given with respect to the alternative allele. References to any previously published UL-GWAS results are given in the six rightmost columns.

**Supplementary Data 6 Functional mapping and annotation (FUMA) of the GWAS SNPs**

FUMA analysis identified 529 lead-SNPs, which mapped to 849 different genes. GWAS SNP P-values were taken from an inverse-variance weighted fixed effects meta-analysis.

**Supplementary Data 7 FUMA gene-set enrichment**

Gene-set enrichment analysis of 849 genes mapped based on GWAS SNPs. The table shows all significant (FDR adjusted P<0.05; Benjamini-Hochberg adjusted one-sided hypergeometric test) pathways enriched across the categories available in the default FUMA analysis.

**Supplementary Data 8 Expression quantitative trait loci (eQTL) for myometrium and ULs**

GWAS SNPs (Supplementary Data 5) were tested for eQTLs for all genes within 500Kbp. Each row includes information for the GWAS SNP being tested (Haplotype Reference Consortium v1.1 imputation panel). The table includes all significant (Benjamini-Hochberg FDR adjusted P<10%, two-sided Wald test) eQTLs. Log-fold-changes are reported with respect to the risk allele.

**Supplementary Data 9 GWAS regions with weaker signal**

Regions with weaker association to UL (p<1e-5, inverse-variance weighted fixed effects meta-analysis). GWAS_location: locus of association signal; SNPs within 500kb distance merged to one region. chrom: chromosome; hg38_position: Hg38 coordinate for the SNP with the most significant association on the region.

**Supplementary Data 10 ChromHMM samples in UL subclass-level segmentations**

Samples used for the subclass-level chromHMM segmentation.

**Supplementary Data 11 List of used ENCODE rE2G links**

ENCODE biosamples used in rE2G analysis.
