# Supplementary material for: Chromatin state origins of uterine leiomyoma
Source: Nat Commun. 2025 May 8;16:4307. doi: 10.1038/s41467-025-59646-w (PMC12062214; doi:10.1038/s41467-025-59646-w)
Supplement: Supplementary file 14 — Reporting Summary [file 41467_2025_59646_MOESM14_ESM.pdf]

nature portfolio

Corresponding author(s): Lauri A. Aaltonen

Last updated by author(s): Apr 28, 2025

Reporting Summary

Nature Portfolio wishes to improve the reproducibility of the work that we publish. This form provides structure for consistency and transparency in reporting. For further information on Nature Portfolio policies, see our Editorial Policies and the Editorial Policy Checklist.

Statistics

For all statistical analyses, confirm that the following items are present in the figure legend, table legend, main text, or Methods section.

n/a	Confirmed	
☐	☒	The exact sample size (*n*) for each experimental group/condition, given as a discrete number and unit of measurement
☐	☒	A statement on whether measurements were taken from distinct samples or whether the same sample was measured repeatedly
☐	☒	The statistical test(s) used AND whether they are one- or two-sided<br>*Only common tests should be described solely by name; describe more complex techniques in the Methods section.*
☐	☒	A description of all covariates tested
☐	☒	A description of any assumptions or corrections, such as tests of normality and adjustment for multiple comparisons
☐	☒	A full description of the statistical parameters including central tendency (e.g. means) or other basic estimates (e.g. regression coefficient) AND variation (e.g. standard deviation) or associated estimates of uncertainty (e.g. confidence intervals)
☐	☒	For null hypothesis testing, the test statistic (e.g. *F*, *t*, *r*) with confidence intervals, effect sizes, degrees of freedom and *P* value noted<br>*Give P values as exact values whenever suitable.*
☒	☐	For Bayesian analysis, information on the choice of priors and Markov chain Monte Carlo settings
☐	☒	For hierarchical and complex designs, identification of the appropriate level for tests and full reporting of outcomes
☐	☒	Estimates of effect sizes (e.g. Cohen's *d*, Pearson's *r*), indicating how they were calculated

Our web collection on statistics for biologists contains articles on many of the points above.

Software and code

Policy information about availability of computer code

Data collection	No software was used for the data collection.
Data analysis	ChIP sequencing<br>Raw sequencing reads were quality and adapter trimmed with cutadapt version 4.1 in Trim Galore version 0.6.7. Trimmed reads were aligned to the GRCh38 reference genome using Bowtie 2 (version 2.5.0) and reads with mapping quality<20 were filtered out with samtools (version 1.6). Peak calling was performed with MACS2 (version 2.2.7.1). 15-state chromatin segmnetations were created with chromHMM (version 1.24).<br><br>NMI clustering<br>Chromatin segmentations were clustered using normalized mutual information (NMI) method in R package aricode (version 1.0.3) and hclust method ward.D2.<br><br>ATAC-seq<br>Reads were quality and adapter trimmed with cutadapt version 1.16 in Trim Galore version 0.3.7. Trimmed reads were aligned to reference genome using Bowtie 2 (version 2.1.0). Samtools (version 1.8) was used to filter out reads with mapping quality<20, count reads mapping to the mitochondrial genome and to remove PCR duplicates. Peak calling was performed with MACS2 (version 2.1.4). Quality was checked with ataqv. Fixed-width peaks were generated using MACS2 callpeak command with parameters '--shift -100 --extsize 200 --nomodel --call-summits --nolambda --keep-dup all -p 0.01'. Differentially accessible regions were calculated with DESeq2 v1.40.2. Enrichment of DARs to myometrium and UL 15-state chromatin segmentations was calculated with Locus Overlap Analysis (LOLA) package32 v.1.30.0. Linear regression was used to calculate the association of Tn5 insertions to genotype of SATB2 risk allele in myometrium ATAC-seq samples using lm-

function in R. ChromVAR (version 1.16) was used to study TF motif accessibility between FH ULs and myometrium.

HiChIP
HiC-Pro v. 3.1.0 was used to identify valid interaction pairs from quality and adapter trimmed reads (Trim Galore v 0.6.7 using Cutadapt v4.1). Bowtie2 v.2.4.4 was used to map reads to reference genome GRCh38. FitHiChIP (version 11.0) was used to call significant interactions and differential links. Differential analysis utilized EdgeR v3.32.1.

CpG methylation
Differentially methylated loci (DML) were determined from sequenced samples with the DSS R package (version 2.28.0) utilizing bsseq R package (version 1.16.1).

Genome-wide association study
Inverse-variance weighted fixed effects meta-analysis (R package "meta" v4.8-4). Functional mapping and annotation of GWAS SNPs were performed using FUMA (v1.5.2) and MAGMA (v1.08). eQTL association tests were performed using DESeq2 (v1.46.0).

For manuscripts utilizing custom algorithms or software that are central to the research but not yet described in published literature, software must be made available to editors and reviewers. We strongly encourage code deposition in a community repository (e.g. GitHub). See the Nature Portfolio guidelines for submitting code & software for further information.

Data

Policy information about availability of data

All manuscripts must include a data availability statement. This statement should provide the following information, where applicable:

- Accession codes, unique identifiers, or web links for publicly available datasets
- A description of any restrictions on data availability
- For clinical datasets or third party data, please ensure that the statement adheres to our policy

The ATAC-seq, RNA-seq, CpG methylation, ChIP-seq against H2A.Z, and HiChIP publicly available data used in this study are available in the European Genome–phenome Archive database under accession code EGAS00001004499 [https://ega-archive.org/studies/EGAS00001004499]4. The 15-state chromatin segmentations and ChIP-seq data generated in this study have been deposited in the Federated European Genome-phenome Archive database under accession code EGAD50000001443 [https://ega-archive.org/datasets/EGAD50000001443]. Due to the sensitive nature of genetic data access is restricted, and can be obtained in FEGA (https://research.csc.fi/service/fega/). Data access requests will be evaluated by the Data Access Committee in accordance with the Finnish legislation and the European General Data Protection Regulation (GDPR), and can be granted to non-commercial academic research on neoplasia. Data requests will be processed in four weeks. Data access period is six months, after which an extension time can be applied. The 15-state chromatin segmentations for myometrium and the UL subclasses can be accessed also through Zenodo [https://doi.org/10.5281/zenodo.13373492]. Differential gene expression data can be accessed in Berta et al. (2021)4 Supplementary Table 21. Used ENCODE rE2G accession numbers are listed in Supplementary Data 11. The previously published GWAS data used in this study are publicly available in Berta et al. (2021)4 Supplementary Table 25; in Sliz et al.
(2023)35 Table 1; and in GWAS Catalog
(https://www.ebi.ac.uk/gwas/downloads, accessed on Sept 9, 2020). Source data are provided with this paper. The remaining data are available within the Article, Supplementary Information or Source Data file.

Research involving human participants, their data, or biological material

Policy information about studies with human participants or human data. See also policy information about sex, gender (identity/presentation), and sexual orientation and race, ethnicity and racism.

Reporting on sex and gender	Findings apply to only one sex. Lack of sex- and gender-based analyses was justified by the phenotype being studied. From the cohort data, we included females based on their genetic sex. For the prospectively collected hysterectomy samples, all participants were females who underwent hysterectomy and had at least one uterine leiomyoma.
Reporting on race, ethnicity, or other socially relevant groupings	No socially constructed variables, or socially relevant categorization variables, were used in the study.
Population characteristics	From the UK Biobank data, we included females of white European ancestry. The FINNGEN and Biobank Japan are cohorts with predominantly Finnish and Japanese ancestry, respectively. For the prospectively collected hysterectomy samples, all participants were females who underwent hysterectomy and had at least one uterine leiomyoma.
Recruitment	The sample set consists of five prospectively collected sample series (My, My1000, My5000, My6000 and My8000). A written informed consent was obtained from all patients. Participants were not compensated. See details of sample collection in the Methods section. Self-selection bias did not affect this study.
Ethics oversight	This study was conducted in accordance with the Declaration of Helsinki and approved by the Ministry of Social Affairs and Health and the National Institute for Health and Welfare (53/07/2000, THL/1071/5.05.00/2011, THL/151/5.05.00/2017, THL/1300/5.05.00/2019, THL/1849/14.06.00/2024), and the Ethics Committee of the Hospital District of Helsinki and Uusimaa (133/E8/03, 408/13/03/03/2009, 177/13/03/03/2016, HUS/2509/2016). Further ethics details of the FinnGen cohort are given in the Methods section.

Note that full information on the approval of the study protocol must also be provided in the manuscript.

Field-specific reporting

Please select the one below that is the best fit for your research. If you are not sure, read the appropriate sections before making your selection.

☒ Life sciences ☐ Behavioural & social sciences ☐ Ecological, evolutionary & environmental sciences

For a reference copy of the document with all sections, see nature.com/documents/nr-reporting-summary-flat.pdf

Life sciences study design

All studies must disclose on these points even when the disclosure is negative.

Sample size	No statistical methods were used to predetermine the sample size.
Data exclusions	ChIP-seq data that did not meet our quality criteria (FRiP>=5 %) were excluded from the study.
Replication	Multiple biological replicates from each UL subclass and normal tissue specimens were included in all analyses. The number of replicates are indicated in each figure legend and/or respective results section.
Randomization	Experimental groups were not used in the study.
Blinding	Sample sizes in ChIP-seq were small and blinding was not possible during DNA processing, library preparations were done blinded. Some data analyses such as clusterings were always done blinded. Blinding was not relevant in analyses comparing subclasses.

Reporting for specific materials, systems and methods

We require information from authors about some types of materials, experimental systems and methods used in many studies. Here, indicate whether each material, system or method listed is relevant to your study. If you are not sure if a list item applies to your research, read the appropriate section before selecting a response.

Materials & experimental systems

n/a	Involved in the study
☐	☒ Antibodies
☒	☐ Eukaryotic cell lines
☒	☐ Palaeontology and archaeology
☒	☐ Animals and other organisms
☒	☐ Clinical data
☒	☐ Dual use research of concern
☒	☐ Plants

Methods

n/a	Involved in the study
☐	☒ ChIP-seq
☒	☐ Flow cytometry
☒	☐ MRI-based neuroimaging

Antibodies

Antibodies used

ChIP-seq:
H3K4me3 (Abcam, Cat. No. ab8580, polyclonal, lot. GR3275503-1,GR3264593-1) 7μg per ChIP.
H3K27me3 (Cell Signaling, Cat. No. 9733S, IgG clone C36B11, lot. 16, 19) 20μl per ChIP.
H3K4me1 (Abcam, Cat. No. ab8895, polyclonal, lot 1017389-1) 7μg per ChIP.
H3K9me3 (Active Motif, Cat. No. 39062/39161, polyclonal, lot 1022004) 10μl per ChIP.
H3K36me3 (Diagenode, Cat. No. C15410058, polyclonal, lot A.8889-001P) 7μg per ChIP.
Spike-in antibody (Active Motif, Cat. No. 61686, lot 4321008) 2μg per ChIP.

Validation

H3K4me1 (Abcam, Cat. No. ab8895): Rabbit Polyclonal H3 mono methyl K4 antibody. Suitable for IHC-P, ICC/IF, ChIP, WB and reacts with Human, Mouse, Rat, Cow samples. Specific for mono-methylated Lysine 4 of histone H3. Does not recognise di- or tri-methyl Lysine 4 nor methylation at Lysine 9. The exact immunogen used to generate this antibody is proprietary information.

H3K9me3 (Active Motif, Cat. No. 39062/39161): Immunogen: This Histone H3 trimethyl Lys9 antibody was raised against a peptide including trimethyl-lysine 9 of histone H3. Applications Validated by Active Motif: ChIP, ChIP-seq, ICC/IF, WB, CUT&Tag.

H3K36me3 (Diagenode, Cat. No. C15410058): Polyclonal antibody raised in rabbit against histone H3, trimethylated at lysine 36 (H3K36me3), using a KLHconjugated synthetic peptide. Specificity: Human, mouse, zebrafish, Drosophila: positive.
Other species: not tested. Applications: ChIP/ChIP-seq, ELISA, Dot Blotting, Western blotting.

H3K4me3 (Abcam, Cat. No. ab8580): Immunogen: Synthetic peptide within Human Histone H3 aa 1-100 (tri methyl K4) conjugated to keyhole limpet haemocyanin. The exact sequence is proprietary. Tested applications: suitable for PepArr, ChIP, WB, IHC-P, ICC/IF. Species reactivity: reacts with Cow, Human; predicted to work with Mouse, Rat, Rabbit, Pig, Saccharomyces cerevisiae, Tetrahymena,

Xenopus laevis, Arabidopsis thaliana, Caenorhabditis elegans, Drosophila melanogaster, Indian muntjac, Oikopleura, Plants, Zebrafish, Mammals, Trypanosoma cruzi, Common marmoset, Rice, Xenopus tropicalis.

H3K27me3 (Cell Signaling, Cat. No. 9733S): “Tri-Methyl-Histone H3 (Lys27) (C36B11) Rabbit mAb detects endogenous levels of histone H3 only when tri-methylated on Lys27. The antibody does not cross-react with non-methylated, mono-methylated or dimethylated Lys27. In addition, the antibody does not cross-react with mono-methylated, di-methylated or tri-methylated histone H3 at Lys4, Lys9, Lys36 or Histone H4 at Lys20.” Species Reactivity: Human, Mouse, Rat, Monkey. Species predicted to react based on 100% sequence homology: Xenopus, Zebrafish.

Spike-in antibody (Active Motif, Cat. No. 61686): “The Spike-in Antibody recognizes a histone variant (H2Av) that is specific to the species of the Spike-in Chromatin (Drosophila). Each lot of Spike-in Chromatin is quantified and tested with the Spike-in Antibody. This enables specific detection of the Spike-in Chromatin without any significant increase in background signal.

Plants

Seed stocks

Report on the source of all seed stocks or other plant material used. If applicable, state the seed stock centre and catalogue number. If plant specimens were collected from the field, describe the collection location, date and sampling procedures.

Novel plant genotypes

Describe the methods by which all novel plant genotypes were produced. This includes those generated by transgenic approaches, gene editing, chemical/radiation-based mutagenesis and hybridization. For transgenic lines, describe the transformation method, the number of independent lines analyzed and the generation upon which experiments were performed. For gene-edited lines, describe the editor used, the endogenous sequence targeted for editing, the targeting guide RNA sequence (if applicable) and how the editor was applied.

Authentication

Describe any authentication procedures for each seed stock used or novel genotype generated. Describe any experiments used to assess the effect of a mutation and, where applicable, how potential secondary effects (e.g. second site T-DNA insertions, mosiacism, off-target gene editing) were examined.

ChIP-seq

Data deposition

☒ Confirm that both raw and final processed data have been deposited in a public database such as GEO.

☒ Confirm that you have deposited or provided access to graph files (e.g. BED files) for the called peaks.

Data access links
May remain private before publication.

Data produced in this work (ChIP-seq peak call bed-files, aligned bam-files and raw reads, and subclass-level chromatin segmentations data can be accessed in the Federated European Genome-phenome Archive (FEGA).

Files in database submission

FH_dense.bed
HMGA2_dense.bed
MED12_dense.bed
My5001N1_H3K27me3.bam
My5001N1_H3K27me3_1.fastq.gz
My5001N1_H3K36me3.bam
My5001N1_H3K36me3_1.fastq.gz
My5001N1_H3K36me3_2.fastq.gz
My5001N1_H3K4me1.bam
My5001N1_H3K4me1_1.fastq.gz
My5001N1_H3K4me1_2.fastq.gz
My5001N1_H3K4me3.bam
My5001N1_H3K4me3_1.fastq.gz
My5001N1_H3K9me3.bam
My5001N1_H3K9me3_1.fastq.gz
My5001N1_H3K9me3_2.fastq.gz
My5001N1_input_H3K27me3_H3K4me3.bam
My5001N1_input_H3K27me3_H3K4me3_1.fastq.gz
My5001N1_input_H3K36me3_H3K4me1_H3K9me3.bam
My5001N1_input_H3K36me3_H3K4me1_H3K9me3_1.fastq.gz
My5001N1_input_H3K36me3_H3K4me1_H3K9me3_2.fastq.gz
My5001T1_H3K27me3.bam
My5001T1_H3K27me3_1.fastq.gz
My5001T1_H3K36me3.bam
My5001T1_H3K36me3_1.fastq.gz
My5001T1_H3K36me3_2.fastq.gz
My5001T1_H3K4me1.bam
My5001T1_H3K4me1_1.fastq.gz
My5001T1_H3K4me1_2.fastq.gz
My5001T1_H3K4me3.bam
My5001T1_H3K4me3_1.fastq.gz

My5001T1_H3K9me3.bam
My5001T1_H3K9me3_1.fastq.gz
My5001T1_H3K9me3_2.fastq.gz
My5001T1_input_H3K27me3_H3K4me3.bam
My5001T1_input_H3K27me3_H3K4me3_1.fastq.gz
My5001T1_input_H3K36me3_H3K4me1_H3K9me3.bam
My5001T1_input_H3K36me3_H3K4me1_H3K9me3_1.fastq.gz
My5001T1_input_H3K36me3_H3K4me1_H3K9me3_2.fastq.gz
My5013T1_H3K27me3.bam
My5013T1_H3K27me3_1.fastq.gz
My5013T1_H3K36me3.bam
My5013T1_H3K36me3_1.fastq.gz
My5013T1_H3K36me3_2.fastq.gz
My5013T1_H3K4me1.bam
My5013T1_H3K4me1_1.fastq.gz
My5013T1_H3K4me1_2.fastq.gz
My5013T1_H3K9me3.bam
My5013T1_H3K9me3_1.fastq.gz
My5013T1_H3K9me3_2.fastq.gz
My5013T1_input_H3K27me3.bam
My5013T1_input_H3K27me3_1.fastq.gz
My5013T1_input_H3K36me3_H3K4me1_H3K9me3.bam
My5013T1_input_H3K36me3_H3K4me1_H3K9me3_1.fastq.gz
My5013T1_input_H3K36me3_H3K4me1_H3K9me3_2.fastq.gz
My5016N3_H3K27me3.bam
My5016N3_H3K27me3_1.fastq.gz
My5016N3_H3K36me3.bam
My5016N3_H3K36me3_1.fastq.gz
My5016N3_H3K36me3_2.fastq.gz
My5016N3_H3K4me1.bam
My5016N3_H3K4me1_1.fastq.gz
My5016N3_H3K4me1_2.fastq.gz
My5016N3_H3K4me3.bam
My5016N3_H3K4me3_1.fastq.gz
My5016N3_H3K9me3.bam
My5016N3_H3K9me3_1.fastq.gz
My5016N3_H3K9me3_2.fastq.gz
My5016N3_input_H3K27me3.bam
My5016N3_input_H3K27me3_1.fastq.gz
My5016N3_input_H3K36me3_H3K4me1_H3K9me3.bam
My5016N3_input_H3K36me3_H3K4me1_H3K9me3_1.fastq.gz
My5016N3_input_H3K36me3_H3K4me1_H3K9me3_2.fastq.gz
My5016N3_input_H3K4me3.bam
My5016N3_input_H3K4me3_1.fastq.gz
My5016T1_H3K27me3.bam
My5016T1_H3K27me3_1.fastq.gz
My5016T1_H3K36me3.bam
My5016T1_H3K36me3_1.fastq.gz
My5016T1_H3K36me3_2.fastq.gz
My5016T1_H3K4me1.bam
My5016T1_H3K4me1_1.fastq.gz
My5016T1_H3K4me1_2.fastq.gz
My5016T1_H3K4me3.bam
My5016T1_H3K4me3_1.fastq.gz
My5016T1_H3K9me3.bam
My5016T1_H3K9me3_1.fastq.gz
My5016T1_H3K9me3_2.fastq.gz
My5016T1_input_H3K27me3.bam
My5016T1_input_H3K27me3_1.fastq.gz
My5016T1_input_H3K36me3_H3K4me1_H3K9me3.bam
My5016T1_input_H3K36me3_H3K4me1_H3K9me3_1.fastq.gz
My5016T1_input_H3K36me3_H3K4me1_H3K9me3_2.fastq.gz
My5016T1_input_H3K4me3.bam
My5016T1_input_H3K4me3_1.fastq.gz
My5018T2_H3K36me3.bam
My5018T2_H3K36me3_1.fastq.gz
My5018T2_H3K36me3_2.fastq.gz
My5018T2_input_H3K36me3.bam

My5018T2_input_H3K36me3_1.fastq.gz
My5018T2_input_H3K36me3_2.fastq.gz
My5019N1_H3K27me3.bam
My5019N1_H3K27me3_1.fastq.gz
My5019N1_H3K36me3.bam
My5019N1_H3K36me3_1.fastq.gz
My5019N1_H3K36me3_2.fastq.gz
My5019N1_H3K4me1.bam
My5019N1_H3K4me1_1.fastq.gz
My5019N1_H3K4me1_2.fastq.gz
My5019N1_H3K4me3.bam
My5019N1_H3K4me3_1.fastq.gz
My5019N1_H3K9me3.bam
My5019N1_H3K9me3_1.fastq.gz
My5019N1_H3K9me3_2.fastq.gz
My5019N1_input_H3K27me3.bam
My5019N1_input_H3K27me3_1.fastq.gz
My5019N1_input_H3K36me3_H3K4me1_H3K9me3.bam
My5019N1_input_H3K36me3_H3K4me1_H3K9me3_1.fastq.gz
My5019N1_input_H3K36me3_H3K4me1_H3K9me3_2.fastq.gz
My5019N1_input_H3K4me3.bam
My5019N1_input_H3K4me3_1.fastq.gz
My5019T1_H3K27me3.bam
My5019T1_H3K27me3_1.fastq.gz
My5019T1_H3K36me3.bam
My5019T1_H3K36me3_1.fastq.gz
My5019T1_H3K36me3_2.fastq.gz
My5019T1_H3K4me1.bam
My5019T1_H3K4me1_1.fastq.gz
My5019T1_H3K4me1_2.fastq.gz
My5019T1_H3K4me3.bam
My5019T1_H3K4me3_1.fastq.gz
My5019T1_H3K9me3.bam
My5019T1_H3K9me3_1.fastq.gz
My5019T1_H3K9me3_2.fastq.gz
My5019T1_input_H3K27me3.bam
My5019T1_input_H3K27me3_1.fastq.gz
My5019T1_input_H3K36me3_H3K4me1_H3K9me3.bam
My5019T1_input_H3K36me3_H3K4me1_H3K9me3_1.fastq.gz
My5019T1_input_H3K36me3_H3K4me1_H3K9me3_2.fastq.gz
My5019T1_input_H3K4me3.bam
My5019T1_input_H3K4me3_1.fastq.gz
My6139T1_H3K27me3.bam
My6139T1_H3K27me3_1.fastq.gz
My6139T1_H3K36me3.bam
My6139T1_H3K36me3_1.fastq.gz
My6139T1_H3K36me3_2.fastq.gz
My6139T1_H3K4me1.bam
My6139T1_H3K4me1_1.fastq.gz
My6139T1_H3K4me1_2.fastq.gz
My6139T1_H3K4me3.bam
My6139T1_H3K4me3_1.fastq.gz
My6139T1_H3K9me3.bam
My6139T1_H3K9me3_1.fastq.gz
My6139T1_H3K9me3_2.fastq.gz
My6139T1_input_H3K27me3_H3K4me3.bam
My6139T1_input_H3K27me3_H3K4me3_1.fastq.gz
My6139T1_input_H3K36me3_H3K4me1_H3K9me3.bam
My6139T1_input_H3K36me3_H3K4me1_H3K9me3_1.fastq.gz
My6139T1_input_H3K36me3_H3K4me1_H3K9me3_2.fastq.gz
My6174T1_H3K27me3.bam
My6174T1_H3K27me3_1.fastq.gz
My6174T1_H3K36me3.bam
My6174T1_H3K36me3_1.fastq.gz
My6174T1_H3K36me3_2.fastq.gz
My6174T1_H3K4me1.bam
My6174T1_H3K4me1_1.fastq.gz
My6174T1_H3K4me1_2.fastq.gz

My6174T1_H3K9me3.bam
My6174T1_H3K9me3_1.fastq.gz
My6174T1_H3K9me3_2.fastq.gz
My6174T1_input_H3K27me3.bam
My6174T1_input_H3K27me3_1.fastq.gz
My6174T1_input_H3K36me3_H3K4me1_H3K9me3.bam
My6174T1_input_H3K36me3_H3K4me1_H3K9me3_1.fastq.gz
My6174T1_input_H3K36me3_H3K4me1_H3K9me3_2.fastq.gz
My6213T1_H3K27me3.bam
My6213T1_H3K27me3_1.fastq.gz
My6213T1_H3K36me3.bam
My6213T1_H3K36me3_1.fastq.gz
My6213T1_H3K36me3_2.fastq.gz
My6213T1_H3K4me1.bam
My6213T1_H3K4me1_1.fastq.gz
My6213T1_H3K4me1_2.fastq.gz
My6213T1_H3K4me3.bam
My6213T1_H3K4me3_1.fastq.gz
My6213T1_H3K9me3.bam
My6213T1_H3K9me3_1.fastq.gz
My6213T1_H3K9me3_2.fastq.gz
My6213T1_input_H3K27me3.bam
My6213T1_input_H3K27me3_1.fastq.gz
My6213T1_input_H3K36me3_H3K4me1_H3K9me3.bam
My6213T1_input_H3K36me3_H3K4me1_H3K9me3_1.fastq.gz
My6213T1_input_H3K36me3_H3K4me1_H3K9me3_2.fastq.gz
My6213T1_input_H3K4me3.bam
My6213T1_input_H3K4me3_1.fastq.gz
My6217T1_H3K27me3.bam
My6217T1_H3K27me3_1.fastq.gz
My6217T1_H3K36me3.bam
My6217T1_H3K36me3_1.fastq.gz
My6217T1_H3K36me3_2.fastq.gz
My6217T1_H3K4me1.bam
My6217T1_H3K4me1_1.fastq.gz
My6217T1_H3K4me1_2.fastq.gz
My6217T1_H3K4me3.bam
My6217T1_H3K4me3_1.fastq.gz
My6217T1_H3K9me3.bam
My6217T1_H3K9me3_1.fastq.gz
My6217T1_H3K9me3_2.fastq.gz
My6217T1_input_H3K27me3_H3K4me3.bam
My6217T1_input_H3K27me3_H3K4me3_1.fastq.gz
My6217T1_input_H3K36me3_H3K4me1_H3K9me3.bam
My6217T1_input_H3K36me3_H3K4me1_H3K9me3_1.fastq.gz
My6217T1_input_H3K36me3_H3K4me1_H3K9me3_2.fastq.gz
Normal_dense.bed
peaks.narrowPeak.allSamples.bed.gz

Genome browser session
(e.g. UCSC)

No longer applicable

Methodology

Replicates

Biological replicates (2-3, see supplementary table 3) were used to create chromHMM segmentations for myometrium and MED12, HMGA2 and FH ULs.

Sequencing depth

H3K4me3 and H3K27me: total reads ; total non-redundant reads3 ChIP-seq samples were sequenced with single-end 100bp reads. H3K9me3, H3K4me1 and H3K36me3 ChIP-seq samples were sequenced with paired-end 150bp reads.

My_5001.1N1_H3K27me3: total reads 29217870 total non-redundant reads 28613908
My_5001.1N1_H3K4me3: total reads 30766780; total non-redundant reads 29924263
My_5001.1N1_H3K4me1: total reads 40393916; total non-redundant reads 37867286
My_5001.1N1_H3K9me3: total reads 34105928; total non-redundant reads 31679568
My_5001.1N1_H3K36me3: total reads 39011046; total non-redundant reads 36388048
My_5001.1T1_H3K27me3: total reads 23720687; total non-redundant reads 22890204
My_5001.1T1_H3K4me3: total reads 24429765; total non-redundant reads 23043692
My_5001.1T1_H3K4me1: total reads 43959622; total non-redundant reads 39741434

My_5001.1T1_H3K9me3: total reads 31943905; total non-redundant reads 30098043
My_5001.1T1_H3K36me3: total reads 43276965; total non-redundant reads 40300725
My_5013.1T1_H3K27me3: total reads 23322373; total non-redundant reads 21106431
My_5013.1T1_H3K4me1: total reads 38089665; total non-redundant reads 35882459
My_5013.1T1_H3K9me3: total reads 32546494; total non-redundant reads 30326370
My_5013.1T1_H3K36me3: total reads 51734040; total non-redundant reads 46421788
My_5016.1N3_H3K27me3: total reads 23641722; total non-redundant reads 22672852
My_5016.1N3_H3K4me3: total reads 19835201; total non-redundant reads 15702325
My_5016.1N3_H3K4me1: total reads 49212685; total non-redundant reads 45314663
My_5016.1N3_H3K9me3: total reads 40622490; total non-redundant reads 37289974
My_5016.1N3_H3K36me3: total reads 45865125; total non-redundant reads 42526801
My_5016.1T1_H3K27me3: total reads 20586101; total non-redundant reads 19626240
My_5016.1T1_H3K4me3: total reads 21573996; total non-redundant reads 17678811
My_5016.1T1_H3K4me1: total reads 41967390; total non-redundant reads 38772910
My_5016.1T1_H3K9me3: total reads 26333399; total non-redundant reads 24699981
My_5016.1T1_H3K36me3: total reads 51682168; total non-redundant reads 46335627
My_5018.1T2_H3K36me3: total reads 40791666; total non-redundant reads 34910106
My_5019.1N1_H3K27me3: total reads 21227604; total non-redundant reads 19933116
My_5019.1N1_H3K4me3: total reads 19414912; total non-redundant reads 18118073
My_5019.1N1_H3K4me1: total reads 48367093; total non-redundant reads 44828315
My_5019.1N1_H3K9me3: total reads 34483981; total non-redundant reads 32055584
My_5019.1N1_H3K36me3: total reads 42300740; total non-redundant reads 39147776
My_5019.1T1_H3K27me3: total reads 21272618; total non-redundant reads 20040145
My_5019.1T1_H3K4me3: total reads 21125749; total non-redundant reads 17621097
My_5019.1T1_H3K4me1: total reads 40019055; total non-redundant reads 37389227
My_5019.1T1_H3K9me3: total reads 26613743; total non-redundant reads 25123453
My_5019.1T1_H3K36me3: total reads 42031300; total non-redundant reads 38785006
My_6139.1T1_H3K27me3: total reads 18179283; total non-redundant reads 16051941
My_6139.1T1_H3K4me3: total reads 17687248; total non-redundant reads 16354770
My_6139.1T1_H3K4me1: total reads 38748116; total non-redundant reads 35934172
My_6139.1T1_H3K9me3: total reads 33768621; total non-redundant reads 31395478
My_6139.1T1_H3K36me3: total reads 36637645; total non-redundant reads 33675879
My_6174.1T1_H3K27me3: total reads 17394946; total non-redundant reads 10300911
My_6174.1T1_H3K4me1: total reads 45047242; total non-redundant reads 41714386
My_6174.1T1_H3K9me3: total reads 32797311; total non-redundant reads 29994586
My_6174.1T1_H3K36me3: total reads 45449527; total non-redundant reads 41694308
My_6213.1T1_H3K27me3: total reads 18509754; total non-redundant reads 16607791
My_6213.1T1_H3K4me3: total reads 17897735; total non-redundant reads 14850552
My_6213.1T1_H3K4me1: total reads 45704523; total non-redundant reads 41617742
My_6213.1T1_H3K9me3: total reads 33164206; total non-redundant reads 31033483
My_6213.1T1_H3K36me3: total reads 42011586; total non-redundant reads 38218601
My_6217.1T1_H3K27me3: total reads 18583363; total non-redundant reads 17008993
My_6217.1T1_H3K4me3: total reads 18057070; total non-redundant reads 13821058
My_6217.1T1_H3K4me1: total reads 46031452; total non-redundant reads 42951877
My_6217.1T1_H3K9me3: total reads 29417934; total non-redundant reads 26772138
My_6217.1T1_H3K36me3: total reads 47070803; total non-redundant reads 42548193

Antibodies

H3K4me3 (Abcam, Cat. No. ab8580, polyclonal, lot. GR3275503-1,GR3264593-1) 7µg per ChIP.
H3K27me3 (Cell Signaling, Cat. No. 9733S, IgG clone C36B11, lot. 16, 19) 20µl per ChIP.
H3K4me1 (Abcam, Cat. No. ab8895, polyclonal, lot 1017389-1) 7µg per ChIP.
H3K9me3 (Active Motif, Cat. No. 39062/39161, polyclonal, lot 1022004) 10µl per ChIP.
H3K36me3 (Diagenode, Cat. No. C15410058, polyclonal, lot A.8889-001P) 7µg per ChIP.
Spike-in antibody (Active Motif, Cat. No. 61686, lot 4321008) 2µg per ChIP.

Peak calling parameters

Trimmed reads were aligned to GRCh38 reference genome using Bowtie 2 and reads with mapping quality<20 were filtered out with samtools (version 1.6) view -q 20. Peaks were called with MACS2 callpeak function using parameters -q 0.01 and input for each biological replicate as a control.

Data quality

Each ChIP included in the study was required to have at least 5% of reads in broadPeaks.

Software

Trim Galore version 0.6.7., Bowtie 2 version 2.5.0, samtools version 1.6, MACS version 2.2.7.1, ChromHMM version 1.24.
